# Phylogenetic plant community structure along elevation is lineage specific

**DOI:** 10.1002/ece3.868

**Published:** 2013-11-08

**Authors:** Charlotte Ndiribe, Loïc Pellissier, Silvia Antonelli, Anne Dubuis, Julien Pottier, Pascal Vittoz, Antoine Guisan, Nicolas Salamin

**Affiliations:** 1 Department of Ecology and Evolution, University of LausanneBiophore, 1015 Lausanne, Switzerland; 2 Swiss Institute of BioinformaticsGenopode, 1015 Lausanne, Switzerland; 3 Division of Biology, Imperial College LondonSilwood Park Campus, Ascot, SL5 7PY, U.K; 4 Institute of Earth Surface Dynamics, University of LausanneGeopolis, 1015 Lausanne, Switzerland

**Keywords:** Community structure, elevation gradient, mountain plants, phylogenetic clustering, phylogenetic overdispersion.

## Abstract

The trend of closely related taxa to retain similar environmental preferences mediated by inherited traits suggests that several patterns observed at the community scale originate from longer evolutionary processes. While the effects of phylogenetic relatedness have been previously studied within a single genus or family, lineage-specific effects on the ecological processes governing community assembly have rarely been studied for entire communities or flora. Here, we measured how community phylogenetic structure varies across a wide elevation gradient for plant lineages represented by 35 families, using a co-occurrence index and net relatedness index (NRI). We propose a framework that analyses each lineage separately and reveals the trend of ecological assembly at tree nodes. We found prevailing phylogenetic clustering for more ancient nodes and overdispersion in more recent tree nodes. Closely related species may thus rapidly evolve new environmental tolerances to radiate into distinct communities, while older lineages likely retain inherent environmental tolerances to occupy communities in similar environments, either through efficient dispersal mechanisms or the exclusion of older lineages with more divergent environmental tolerances. Our study illustrates the importance of disentangling the patterns of community assembly among lineages to better interpret the ecological role of traits. It also sheds light on studies reporting absence of phylogenetic signal, and opens new perspectives on the analysis of niche and trait conservatism across lineages.

## Introduction

Two main ecological processes are widely recognized to govern the assembly of communities from a regional species pool. First, neutral processes, which encompass demographic stochasticity coupled with dispersal limitations and community drift, create random patterns of species coexistence (Hubbell 2001; Chave 2004). Second, niche-based processes emerge from the functional or physiological traits that mediate species tolerance to environmental conditions and interspecific competition (MacArthur and Levins 1967; Weiher et al. 2011). The latter processes has long been thought to be influenced by the pattern of shared ancestry existing between species (Cavender-Bares et al. 2009; Vamosi et al. 2009) and will be particularly strong if the evolution of niche traits are conserved along phylogenetic lineages (Webb et al. 2002; Mayfield and Levine 2010). Phylogenetic relatedness has therefore been proposed as a useful additional tool to better understand the drivers of community assembly (Webb et al. 2002; Losos 2008).

Indeed, the recent, large interest in investigating phylogenetic niche conservatism (Wiens and Graham 2005; Wiens et al. 2010) in a wide range of organisms such as plants (Webb 2000; Silvertown et al. 2001), birds (Graham et al. 2009), and lizards (Losos et al. 2003) has paralleled the use of phylogenetic relatedness to detect patterns of community assembly (Webb et al. 2002; Cavender-Bares et al. 2009). In this context, patterns of phylogenetic clustering are expected when environmental filters drive species assemblages to comprise closely related species, and are plausible only under the assumptions of phylogenetically conserved traits (Webb 2000; Vamosi et al. 2009). On the other hand, patterns of phylogenetic overdispersion are generally inferred when niche differentiation or ecological fitting mediates community assembly (Janzen 1985; Cavender-Bares et al. 2004; Bryant et al. 2008). Overdispersion can result either from competition or facilitation, if species traits are conserved along the phylogeny, or environmental filtering and if convergence dominates the evolution of ecologically important traits (Cavender-Bares et al. 2009; Swenson and Enquist 2009; Mayfield and Levine 2010). Investigating the phylogenetic structure of communities, in terms of clustering and overdispersion, can therefore provide insights into how intrinsic and extrinsic factors influence the structure of ecological communities by favouring some lineages over the other (Vamosi et al. 2009).

While in some lineages, ecological traits may be phylogenetically conserved, in others, they may show high evolutionary lability (Cavender-Bares et al. 2004), which has been an important process in several examples of radiation allowing the occupancy of divergent niches. This is particularly visible in lineages that radiate into contrasted environmental conditions such as observed, for example in the Caribbean Anolis lizards (Losos et al. 2003). At the other extreme, considering a wide taxonomic range within the angiosperms may lead to the absence of community phylogenetic patterns (Silvertown et al. 2006), because lineages of very different ages whose evolution might have been governed by very different processes are analyzed jointly. In contrast, examining phylogenetic patterns within a specific taxonomic group is likely to reveal more distinct assembly patterns (Swenson et al. 2006; Hardy and Senterre 2007; Losos 2008), as shown for the Floridian oaks (Cavender-Bares et al. 2004). These studies give evidence that niche conservatism may not be a universal rule of evolution across all taxa (Cavender-Bares et al. 2004; Losos 2008), but, that in fact, lineage-specific differences in the evolution of traits should be expected (Smith and Donoghue 2008), as well as differences between traits within the same taxa (Parra et al. 2010). They have prompted proposals to adopt a lineage-based perspective to investigating the way communities are structured (e.g., Hardy and Senterre 2007; Losos 2008; Vamosi et al. 2009; Parra et al. 2010), because it can better distinguish delicate patterns of differential species’ evolution and variations within and among communities.

The forces governing community assembly and how this may relate to the phylogeny do not solely vary across lineages, but also change along environmental gradients. This implies that neutral assembly, competition, and environmental filtering may prevail under distinct environmental conditions (Graham et al. 2009). For instance, patterns similar to phylogenetic niche conservatism were identified in microbial communities along an elevation gradient, while plant species were found to be less conserved at higher elevations than lower elevation (Bryant et al. 2008). Graham et al. (2009) showed more clustered bird communities at high elevation, possibly indicating a greater prevalence of environmental filtering in harsh abiotic conditions. Despite adopting a lineage-specific approach to assessing community assembly (Parra et al. 2010, 2011; Duarte et al. 2012), no study so far has, to our knowledge, revealed the trends of plant phylogenetic community structure within a lineage-specific approach across a wide elevation gradient of the Alps.

Furthermore, most studies of phylogenetic community assembly have been conducted in tropical regions (e.g., Webb 2000; Hardy and Senterre 2007), the neotropics (e.g., Kembel and Hubbell 2006; Swenson and Enquist 2009), and temperate lowlands (e.g., Silvertown et al. 2001, 2006; Cavender-Bares et al. 2004), while ecosystems along larger elevation gradients have not been considered so far. Not only do mountainous areas classically depict archipelagos (Körner 2003), with varying functional structures along the climatic cline (Theurillat et al. 2003; Pellissier et al. 2010a), but the interplay of topographically complex alpine areas with past climate change has contributed to a rich phylogenetic history through multiple events of sympatric and allopatric speciation.

In this study, we assess lineage-specific phylogenetic community patterns within angiosperms along a broad elevation gradient in a temperate mountain range. We used a comprehensive community-level sampling of an entire regional flora, containing all of the most abundant species as well as species-level phylogenetic information. This provided the necessary context to assess the processes leading to community assembly in mountain environments. In particular, we tested three main hypotheses:

H_1_: When considering most angiosperm species in a mountain flora, the evolutionary development and history of the different lineages may not reflect relevant ecological differences (Losos 2008), because many lineage splits occurred millions of years ago under different ecological conditions (Körner 2003). As a consequence, we expect no overall trend between species co-occurrence and phylogenetic distances. We evaluate this hypothesis using an index of species co-occurrence published in Pellissier et al. (2010b).

H_2_: Because ecological communities tend to show higher niche conservatism at large spatial and phylogenetic scales (Swenson et al. 2006), we expect lineage-specific patterns of community assembly to be phylogenetically clustered at older nodes, but show more opposing patterns (i.e., overdispersion or neutral) within younger lineages. We evaluate this hypothesis using net relatedness index (NRI) because it is appropriate for measuring the overall phylogenetic relatedness among species in a community (Kembel et al. 2010).

H_3_: Phylogenetic diversity may decrease with elevation in most angiosperm lineages due to harsh cold environmental conditions; thus, we expect the elevation and associated environmental conditions to drive more clustering among angiosperm lineages with higher tolerance to conditions at higher elevation. We evaluate this hypothesis by relating NRI to elevation using linear regressions.

## Materials and Methods

### Study site

We surveyed a 700 km^2^ study area in the Swiss Western Alps (6°50′–7°10′E, 46°10′–46°30′N). The elevation of the area ranges from 375 m to 3200 m above sea level. Mean annual temperature and precipitation vary, respectively, from 8°C and 1200 mm at 600 m to −5°C and 2600 mm at 3000 m (Bouët 1985). The soil parent material is predominantly calcareous. Anthropogenic activities such as livestock grazing have a major influence on vegetation structure. This occurs predominantly at lower elevations, owing to easier accessibility to these areas (Randin et al. 2006). In this study, we considered only open nonforested areas (e.g., meadows, pastures, and rocky areas) within the elevation gradient.

### Community sampling

Species were inventoried in plots selected with a random stratified sampling strategy based on elevation, slope, and aspect (Hirzel and Guisan 2002). These plots were distant enough (at least 200 m) to minimize spatial autocorrelation in species occurrences. The field sampling spanned the summers of 2002–2010 with abundance data recorded from a total of 912 2 × 2 m^2^ vegetation plots. Species abundances were estimated from the species cover in eight classes following (Vittoz and Guisan 2007): <0.1%, 0.1–1%, 1–5%, 5–15%, 15–25%, 25–50%, 50–75%, and >75%. For our analyzes, we used the mean values of these classes: 0.05, 0.5, 3, 10, 20, 37.5, 62.5, and 82.5%. Based on the classes, a total of 231 of 260 most abundant angiosperm species (present more than 20 times in the vegetation plots dataset) in 131 genera and 35 plant families were retained to describe the community structure. These species were collected for DNA extraction and sequencing.

### DNA extraction and sequencing

Total DNAs were extracted from silica-dried leaf materials collected from the study area, using Qiagen's DNA kit (Qiagen, Hilden, Germany). CTAB protocols (Doyle 1987) were used for about 14 species with strong chemical inhibiting compounds. To ensure thorough pulverization, leaf samples were ground with 2 pellet balls using a standard pulverizing machine for 60 sec and 30 Hz. The plastid *rbcL* gene (ribulose-1,5-bisphosphate carboxylase/oxygenase large subunit) was amplified by polymerase chain reaction (PCR) using standard forward and reverse primers for angiosperm species (Olmstead et al. 1992). Reactions were performed on ice in 50 μL volumes, each containing 33.6 μL of sterile water, 10.0 μL of 10× DNA polymerase buffer 3.0 μL of dNTP (10 mmol/L), 1.0 μL of each primer (10 μmol/L), 0.4 μL of *Taq* DNA polymerase (Bioline, London, UK), and 1.0 μL of aqueous dilution of DNA. PCR amplification was carried out on an Applied Biosystems GeneAmp 2700 thermal cycler (Applied Biosystems, Foster City, CA) using an initial denaturation of 2 min at 94°C followed by 34 cycles of 60 sec at 94°C, 60 sec at 50°C of annealing time, 2 min at 72°C of extension, and a final extension of 7 min at 72°C. Resultant PCR products were run on 1.2% agarose gels and stained with ethidium bromide before viewing in GeneSnap (Syngene, Frederick, MD). They were purified using a Qiaquick PCR purification kit (Qiagen) before the sequencing reaction. Cycle sequencing of the purified PCR products was performed with the forward and reverse primers in both directions, and an additional primer 20R (5′-TGCATTGC [A/G] CGGTG [A/G] ATGTG-3′) was designed to capture most of the internal part of the *rbcL* gene. Reactions were performed on ice in 10 μL volumes, each contained 5.0 μL of sterile water, 2.0 μL of sequence terminator ABI Big Dye version 3.1 (Applied Biosystems; sequencing kit manual), 1.0 μL of each primer, and 2.0 μL of cleaned PCR product. Sequencing reaction was carried out on an initial denaturation of 3 min at 96°C, 30 cycles of 15 sec at 96°C, 15 sec at 50°C, and 90 sec at 60°C. Cycle sequencing products were visualized on an ABI 3100 DNA sequencer (Applied Biosystems). Sequences were first checked for identity by BLAST search for highly similar sequences using the NCBI online blast facility. Forward and reverse contigs were edited and assembled with DNA Baser version 3.x (DNA Baser; Heracle Biosoft, Pitesti, Romania, 2010) before exported for alignment. We augmented the data to 231 species sequences by downloading 73 *rbcL* and 123 *matK* sequences from published sources available in GenBank (Table S1). All sequences generated as part of this study, and the alignments have been deposited in Dryad (doi:10.5061/dryad.q0fh6734) and in GenBank (accession numbers KF602071-KF602251).

### Phylogenetic analyses

Alignment of *rbcL* and *matK* sequences within each plant family was performed with Clustal W algorithm in Mega (Tamura et al. 2007) and Seaview (Gouy et al. 2010). Profile alignment was used to align sequences between families before manual inspection of the final alignment. The final *rbcL* and *matK* matrix consisted of 3092 nucleotides in 231 species. *Abies alba* Mill*.,* and *Picea abies* (L.) H. Karst were included as outgroup species. The best model of DNA substitution was tested using jModelTest (Posada 2008), which resulted in the selection of the GTR + Γ for both DNA regions. Bayesian inference (BI) was performed in MrBayes (Huelsenbeck and Ronquist 2001) using the selected model. The prior distributions relied on four Markov chain Monte Carlo (MCMC) chains of 30 million generations sampling species every 1000 generations. Convergence of the independent run was assessed by checking the log-likelihood and sampled model parameters in Tracer (Drummond and Rambaut 2007). The initial 10,000 trees were discarded, leaving 20,000 trees for estimation of the maximum clade credibility consensus tree.

Estimation of divergence times was performed with Beast (Drummond and Rambaut 2007) with the GTR + Γ model of evolution. Specifically, nine fossils obtained from the study by Magallon and Castillo (2009) (Table 1) were used as minimal age constraints for plant stem (Brassicaceae and Polygonaceae) and crown groups (Apiales, Dipsacales, Ericales, Malpighiales, Rosaceae, eudicots, and angiosperms). The searches were run assuming an uncorrelated lognormal relaxed molecular clock and Yule process for speciation rates. The calibration points took a lognormal distribution (Table 1) with the means and standard deviation chosen to reflect our confidence in the fossils used. The MCMC chain was run for 80 million generations, with trees sampled every 1000 generations. Convergence was also assessed in Tracer by checking the effective sample size (ESS) of the model parameters and assessing the stability of posterior probabilities on individual nodes from the 95% highest posterior density (HPD) estimates (e.g., Rabosky et al. 2011). The first 40,000 trees were discarded as burn-in, before reconstructing the molecular dated tree. The resulting phylogenetic trees were checked against the Angiosperm Phylogeny Group tree for accepted relationships among plant orders and families (APG III Group 2009). The outgroup species were removed from the calibrated tree to perform all the subsequent analyses.

**Table 1:** Plant fossils used in molecular phylogenetic tree calibration. Plant families from this study are placed in parentheses within respective plant orders. A log-normal distribution (mean = 1.0, standard deviation = 0.1) was used for each fossil calibration. The prior distributions on fossil calibration only differed by the offset used.

Calibration point	Fossil	Fossil age (Mya) used as offset	Hard lower bound/mean/soft upper bound (95%; Mya)
CG	Angiosperms	130	132.2/132.7/133.3
CG	Eudicots	125	127.2/127.7/128.3
CG	Apiales	37.2	39.4/39.9/40.5
SL	Brassicaceae	89.3	91.5/92.0/92.6
CG	Dipsacales (Dipsacaceae, Valerianaceae)	33.9	36.1/36.6/37.2
CG	Ericales (Ericaceae, Primulaceae)	89.3	91.5/92.0/92.6
CG	Malpighiales (Euphorbiacea, Hyperiaceae, Linaceae, Salicaceae, Violaceae)	89.3	91.5/92.0/92.6
SL	Polygonaceae	5.33	7.6/8.0/8.6
CG	Rosaceae	37.2	39.4/39.9/40.5

Source: Magallon and Castillo (2009), CG, crown; SL, stem groups.

### Phylogenetic community structure

To evaluate our three hypotheses, we only included the 693 plots in which the 231 selected species accounted for at least 80% of the relative vegetation cover (see Table S2). This was performed to ensure a stronger representation of patterns in the regional pool, based on the global and fine-scale analyses of community phylogenetic structure.

### Global analysis of phylogenetic community structure (H_1_)

To test our first hypothesis (H_1_), that no overall trend will be detected between co-occurrence and phylogenetic distances, we assessed the global phylogenetic structure of the regional pool by matrix correlations between pairwise phylogenetic distances and species co-occurrences, and assessed the significance using Spearman's correlations and 9999 randomizations. In order to account for taxonomic and spatial scale in this analysis, we measured phylogenetic structure within two separate groups consisting of monocots and eudicots, as well as in each vegetation zone (i.e,. colline, montane, subalpine, and alpine, except the nival) along elevation.

The co-occurrence calculation was based on a simple algorithm that estimates the degree to which pairs of species co-occur within communities (Pellissier et al. 2010b). For each pair of species (S1 and S2), the number of communities where both species are present is weighed by the number of communities where the rarer of the two species is present. This index ranged from 0 to 1 (0 = no co-occurrence and 1 = complete co-occurrence) as given in equation 1:


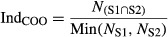
(1)

where *N*_(S1∩S2)_ is the number of times species S1 and S2 co-occur, while Min (*N*_S1_*, N*_S2_) is the occurrence frequency of the rarer of the two species (Pellissier et al. 2010b).

Phylogenetic signal in co-occurrence is interpreted from a significant negative or positive correlation between phylogenetic distances and co-occurrences, which signifies phylogenetic clustering or overdispersion, respectively (Cavender-Bares et al. 2004). Thus, finding a correlation between phylogenetic distances and co-occurrences indicates that communities exhibit a phylogenetic structure as measured by an index of phylogenetic structure such as NRI.

### Lineage-specific analysis of phylogenetic community structure (H_2_)

To test our second hypothesis (H_2_), that communities are more clustered at older nodes, we computed the NRI for each of the 693 communities using the package Picante (Kembel et al. 2010) in conjunction with the Geiger library (Harmon et al. 2008). NRI is the same as the negative standardized effect size mean pairwise phylogenetic distance (MPD) among species in a community and is given in equation 2 (Webb et al. 2002; Kembel et al. 2010):


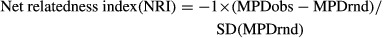
(2)

where “obs” is the observed community, “rnd” is the random community and “SD” is the standard deviation (Webb et al. 2002).

We chose this index because it is sensitive to phylogeny-wide patterns, and the computation of phylogenetic structure explicitly provides the statistical power to unravel the dominant phylogenetic pattern in a community (Webb et al. 2002; Kembel et al. 2010). The calculation was based on subtree phylogenetic distances in each community, present at each node, tested against 9999 null communities. The null model randomizations were based on random shuffling of taxa within the set of taxa present in a given community, while maintaining species richness and prevalence (Kembel and Hubbell 2006; Parra et al. 2011). This ensured that the NRI was only influenced by the species pool that subtend from the node of interest. A total of 230 phylogenetic tree nodes were estimated (except for terminal nodes with only two species). The number of communities at each node that was used to estimate average NRI values is provided in Table S2. We estimated average NRI, along with the deviation from the expected null distribution (i.e., the standard deviation of the mean NRI at each node), separately for all of the species descending from each phylogenetic tree node. These values were then plotted on the phylogenetic tree to distinguish the main trend at each node. Average NRI values of 71 (30.9%) nodes with only two species could not be estimated during the analyses, since NRI is an effect size measure that relies on more than one comparison. Positive values of NRI indicate phylogenetic clustering, while negative values indicate phylogenetic overdispersion (Webb 2000).

We also related average NRI to node ages, to better distinguish how the time scale on a phylogeny may affect the detection of phylogenetic patterns across a particular species pool. As variations in the species richness of lineages may create a methodological bias in the level of relatedness detected between species contained in that lineage (Webb 2000), we used spearman's rank correlations to determine whether the species richness of lineages had a significant correlation with the patterns of phylogenetic assembly at each node. Finally, we measured the effects of lineage age and the community size (or species richness) at nodes on average NRI using a generalized linear model (GLM); this analysis was performed to differentiate the influence of age from the phylogenetic patterns observed at each node.

### Lineage-specific analysis of phylogenetic community structure along elevation (H_3_)

To test our third hypothesis (H_3_), that a higher decrease of phylogenetic patterns among angiosperm lineages occurs with elevation, we assessed the relationship between NRI and elevation for each phylogenetic tree node using linear regressions. The *t*-statistic of the slope coefficient of the regression analyses was then projected on each node of the phylogenetic tree. The significance of this relationship was assessed directly from the regression summary. Linear regressions were used because they provide a simple metric and contain the data output of interest for example estimates of coefficients and 95% prediction interval bands. In addition, the uniformity in sampling efforts across our study area rendered this analysis appropriate for our questions.

Lastly, we assessed the consistency of the phylogenetic assembly patterns by repeating the analyses on 100 randomly sampled trees from the posterior distribution of trees generated in Beast. This was performed to give an indication of the overall uncertainty in our estimate of the average NRI values. All statistical analyses were conducted in the R programming environment.

## Results

### Phylogenetic analyses

The phylogenetic reconstructions, including divergence time estimations, produced well-supported phylogenetic trees with nodes congruent to taxonomic groups defined by the APG III Group (2009) classification and previous findings (e.g., Magallon and Castillo 2009). A total of 56% of the nodes in the dated tree had 100% bootstrap support. A total of 77% nodes had posterior probabilities greater than 90% (Fig. S1). In general, only a few nodes showed low support values, such as the placement of *Cirsium spinosissimum* (L.) Scop. and *Cirsium oleraceum* (L.) Scop. within the Asteraceae family, and some nodes within the Cyperaceae and Lamiaceae family.

### Phylogenetic community structure

#### Global analysis of phylogenetic community structure (H_1_)

**Figure 1:**
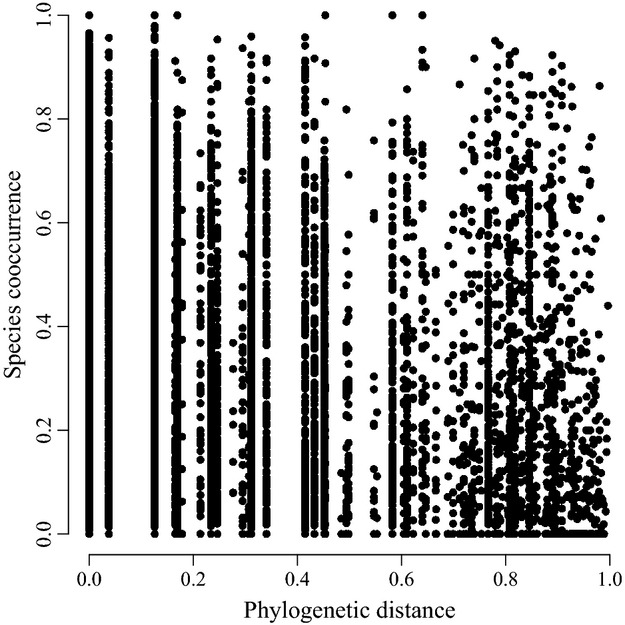
The global relationship between species co-occurrences and phylogenetic distances. This relationship assessed by randomization tests was not significantly different from random (*r* = −0.0014, *P* = 0.47). Species co-occurrence (0 = no co-occurrence, 1 = complete co-occurrence).

The global relationship between phylogenetic distance and species co-occurrence for angiosperm lineages was not significantly different from zero (*r* = −0.0014, *P* = 0.47; Fig. 1), which is congruent with our first hypothesis (H_1_) that considering different angiosperm lineages together could result in the absence of a detectable community structure. Similar results were obtained within the monocot (*r* = −0.08, *P* = 0.85) and eudicot (*r* = −0.01, *P* = 0.60) lineages (Fig. S2), as well as for the communities in each vegetation zone: colline (*r* = 0.004, *P* = 0.45), montane (*r* = −0.004, *P* = 0.50), subalpine (*r* = 0.01, *P* = 0.29), and alpine (*r* = −0.003, *P* = 0.55) along elevation (Fig. S3).

#### Lineage-specific analysis of phylogenetic community structure (H_2_)

**Figure 2:**
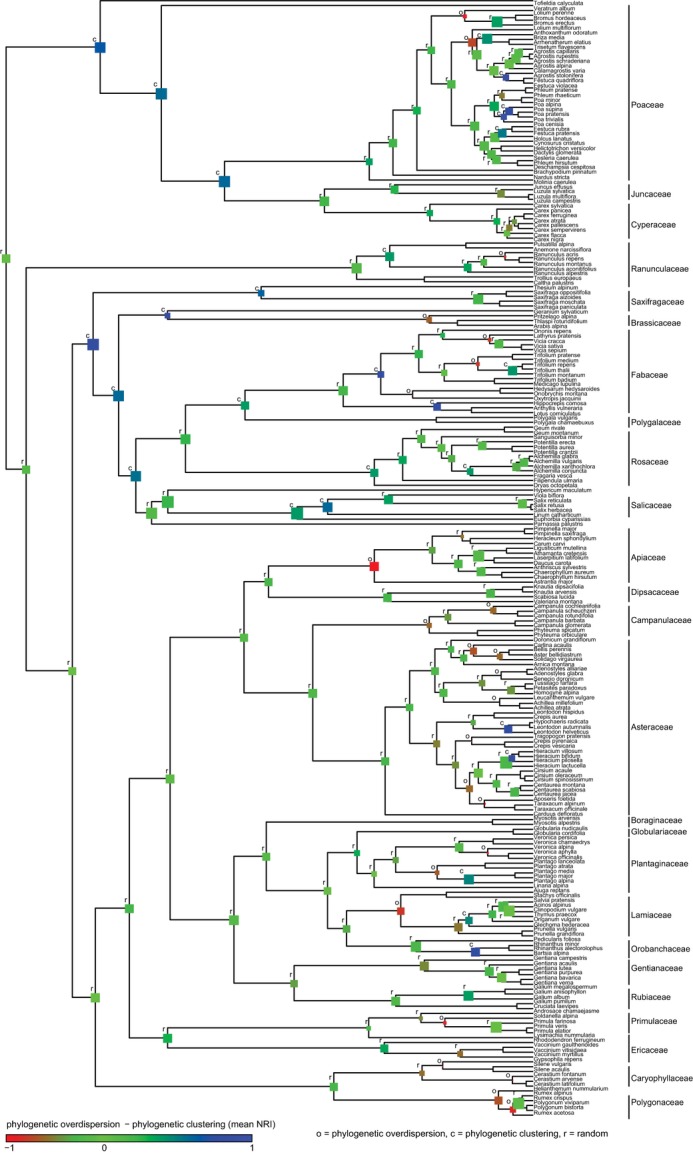
Lineage-specific community assembly at 230 phylogenetic subtree nodes, the observed patterns represents the average net relatedness index of 693 local communities in the Western Swiss Alps. Phylogenetic overdispersion = red, phylogenetic clustering = blue, and no phylogenetic trend = green. The different sizes of the squares represent the standard deviation of the phylogenetic patterns from the mean. o, phylogenetic overdispersion; c, phylogenetic clustering; r, random.

Following our second hypothesis (H_2_), lineage-specific community assembly revealed both patterns of phylogenetic clustering and overdispersion, as well as no observable trend for various lineages (Fig. 2). More opposite phylogenetic patterns emerged toward the tips of the phylogenetic tree than in the basal nodes. Of the 230 nodes, 159 nodes were estimated. Since positive values of NRI indicate phylogenetic clustering, whereas negative values indicate phylogenetic overdispersion (Webb 2000), on a scale of 0–1 and 0 to −1, the average NRI per node revealed phylogenetic clustering in 82 (51.6%) nodes of the phylogenetic tree and phylogenetic overdispersion in 77 (48.4%) nodes. However, to detect random patterns of assembly, we defined average NRI between −0.5 and 0.5 (i.e., based on the 95% CI of the average NRI), values below and above represented phylogenetic overdispersion and phylogenetic clustering, whereas intermediate values represented random patterns. Based on this, a total of 27 (16.9%) nodes were phylogenetically clustered, 25 (15.7%) nodes were phylogenetically overdispersed, whereas 107 (67.3%) nodes showed weak pattern or no trend. Most of the phylogenetically clustered nodes and nodes with no trend showed a larger variance in the respective average NRI values, in contrast to the phylogenetically overdispersed nodes. The mean variance for phylogenetically clustered nodes and nodes with no trend were 0.93 and 0.91 respectively, while it was 0.48 for phylogenetically overdispersed nodes.

Overall, the two most clustered nodes were nodes 211 (*Poa cenisia, Poa pratensis, Poa supina* and *Poa trivialis*) and 220 (*Agrostis stolonifera, Festuca quadriflora* and *Festuca violacea*), whereas the two most overdispersed nodes were nodes 23 (*Primula elatior, Primula farinosa, Primula veris* and *Soldanella alpina*) and 228 (*Bromus erectus, Bromus hordeaceus, Lolium multiflorum* and *Lolium perenne*; Fig. 2). Further, Spearman's rank correlation for each of the 230 nodes showed that NRI and species richness were only significantly correlated with 18 (7.8%) nodes (this was 16% of the 113 nodes estimated; see Fig. S4).

#### Lineage-specific analysis of phylogenetic community structure along elevation (H_3_)

**Figure 3:**
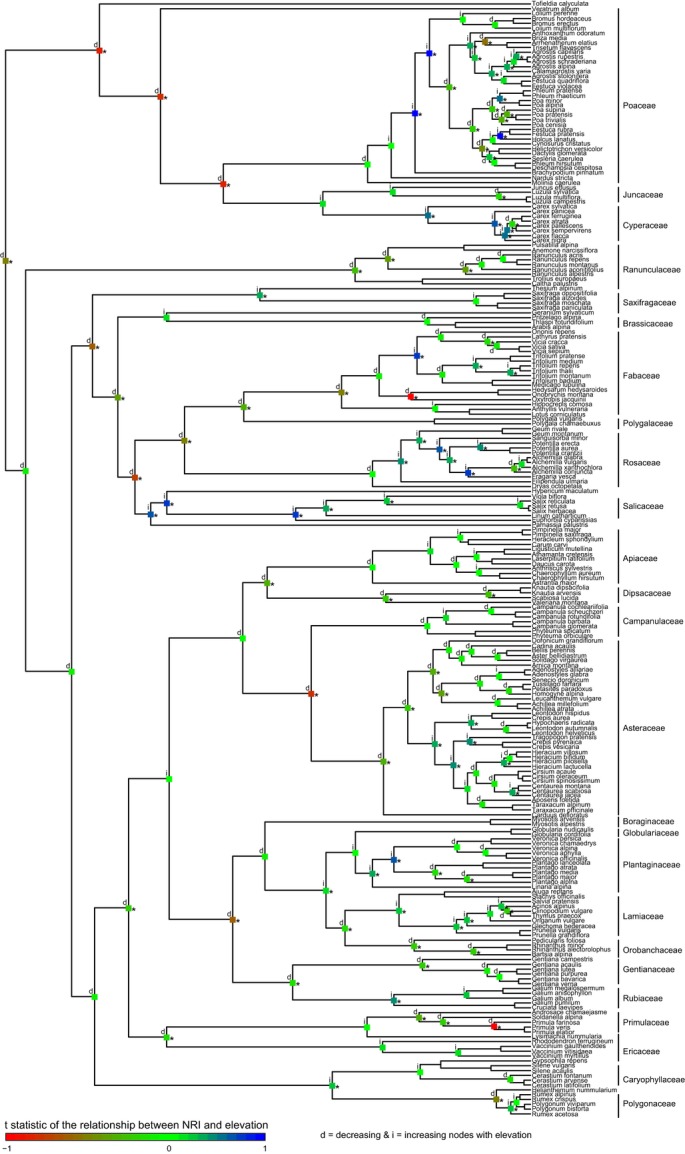
The *t*-statistic of the relationship between net relatedness index (NRI), and elevation at phylogenetic tree nodes. The subtree nodes show a transition of NRI from decrease to increase. Decrease in NRI (red), no overall trend (green), and increase in NRI (blue) with elevation. The stars on nodes indicate a significant decrease or increase in NRI with elevation. d, decreasing; i, increasing nodes with elevation.

For our third hypothesis (H_3_), the relationship between NRI and elevation showed no strict pattern when considering all angiosperms together (Fig. 3). Rather we observed different effects of elevation on angiosperm lineages from the various nodes of the phylogenetic tree. NRI along elevation was found to decrease in 85 (53.8%) nodes, whereas it increased in 73 (46.2%) nodes with increasing elevation (Fig. 3). Among these nodes, significant decrease in NRI with elevation occurred in 48 (56.5%) nodes, while there was significant increase in 45 (61.6%) nodes. Overall, the two nodes that showed the most significant decrease with elevation were nodes 24 (*Primula elatoir, Primula farinosa* and *Primula veris*) and 154 (*Hedysarum hedysaroides, Onobrychis montana* and *Oxytropis jacquinii*), whereas the two nodes with the most significant increase with elevation were nodes 198 (*Agrostis alpina, Agrostis capillaries, Agrostis rupestris,* etc.) and 199 (*Agrostis alpinas, Agrostis capillaris, Agrostis rupestris,* etc.; Fig. 3). Some lineages were significantly more clustered, for example, Cyperaceae species, or more overdispersed, for example Polygonaceae species, with higher elevation, while others largely showed no significant trend, for example Apiaceae species. In total, the NRI of 65 (41.1%) subtree nodes showed no significant trend with elevation.

**Figure 4:**
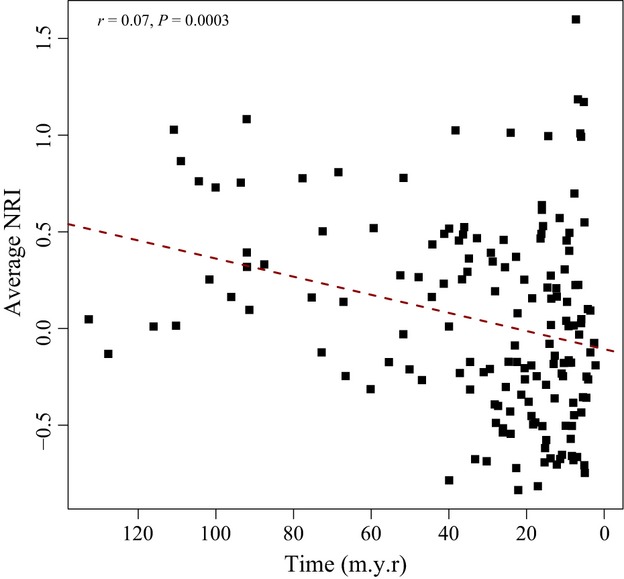
Relationship between average net relatedness index and lineage divergence times (node ages) shows that the characterization of phylogenetic structure may be partly related to the age of lineages. The red dashed line corresponds to a linear regression.

Furthermore, we found the association between average NRI and node ages to significantly increase with evolutionary time (Fig. 4). While this is a likely indication that the appearance of phylogenetic patterns in a community may be dependent on the age of lineages considered, we illustrate a decreasing trend where ancient nodes are more phylogenetically clustered and younger nodes more overdispersed. The significant relationship between NRI and lineage age without a positive effect of community size suggests that NRI and the phylogenetic patterns at nodes are not likely a statistical artifact that arise from the measurement of this index at various scales (Table 2). The slope values summarized across 100 randomly sampled trees from the posterior distribution of dated trees showed very little variation compared with that estimated over the consensus tree (Fig. S5).

**Table 2:** Summary coefficients of the generalized linear regression model used to discriminate the effect of community size and lineage age on observed phylogenetic patterns, interpreted from average net relatedness index (NRI). Community size showed a significant but negative trend, while node age showed a significant and positive trend with average NRI at nodes.

	Estimate	*P*-value
Intercept	–0.139	0.012
Community size	–0.004	0.021
Node age	0.008	2.94e-05

## Discussion

Overall, we found no phylogenetic pattern within angiosperm communities when considering all species together, but rather prevailing phylogenetic clustering for more ancient nodes and overdispersion in more recent tree nodes. These results shed light on previous studies reporting absence of phylogenetic signal in communities, and open new perspectives on how to analyze niche and trait conservatism across lineages. We discuss these results and their implications in the following sections.

### Why don't we see an overall phylogenetic signal (H_1_)?

When considering all species together, community assembly was not strongly structured as regards the phylogeny as indicated by the absence of signal detected in the global analysis between phylogenetic distances and co-occurrences. A similar absence of phylogenetic signal in communities was reported for two meadow communities segregating along a hydrological gradient and which also contained a broad taxonomic range of species (Silvertown et al. 2006).

Phylogenetic structure is usually interpreted from the measures of co-occurrence patterns estimated globally over a phylogenetic tree, as well as the deviations of phylogenetic diversity from null expectations (Vamosi et al. 2009; Kembel et al. 2010). Hence, a detection of no phylogenetic signal in a community could be because: (1) opposite forces of phylogenetic clustering and overdispersion act simultaneously and cancel out (Kembel and Hubbell 2006; Mayfield and Levine 2010); (2) historical contingency and dispersal dominate most community interactions (Hubbell 2001); (3) species niches or traits are more phylogenetically random than patterned (Kembel and Hubbell 2006); and (4) thus, phylogenetic relationships do not reflect the ecological differences among groups of species (Losos 2008). Thus, using phylogenetic relatedness as ecological distance when comparing taxa that diverged under entirely different ecological conditions may therefore not be relevant (Weiher and Keddy 1999). In this case, different lineages would have evolved different new life histories, which render them less comparable with time (Fig. 2). Alternatively, it could be that more recent nodes omit more of the taxa in communities and thus eliminate several strong signals of phylogenetic community structure. Lastly, the statistical power associated with the pairwise randomization analysis may have reduced the detection of phylogenetic signal. However, this is rather unlikely given the large sample size of our data set.

### Why a lineage-specific approach to community assembly (H_2_ and H_3_)?

A more detailed approach as provided by the lineage-specific analyses (i.e., within subtrees) can help unravel the ecological structure of communities at fine taxonomic scales (Hardy and Senterre 2007; Losos 2008). Using such an approach, we found several contrasting patterns of assembly in different angiosperm lineage communities. However, the prevalence of random patterns across the phylogeny may indicate that (1) most communities are structured by neutral processes (Hubbell 2001; Kraft et al. 2007); (2) phylogenetic relatedness may not be a suitable indicator of ecological distances in these lineages, because the inherent ecological traits of the species are not conserved (Cahill et al. 2008); and (3) ecological forces of environmental filtering and niche differentiation counteract each other, resulting in opposing interactions, which create apparent neutral effects in these communities (Cavender-Bares et al. 2009). We believe that the latter is more likely given the two prevailing deterministic phylogenetic patterns found across the phylogeny.

While the simultaneous comparison of angiosperm lineages may not reflect relevant ecological differences (Silvertown et al. 2006), given the evolutionary time interval and different ecological conditions under which many lineages split (see APG III Group 2009), lineage-specific assembly, however, tends to reveal more distinguishing patterns (i.e., clustering, overdispersion, or neutral) of community assembly. Furthermore, lineage-specific community assembly may provide additional insights into patterns created by lineage diversification, the mode of speciation producing ecologically similar or divergent species, and the dynamics controlling niche occupancy and niche exploration (McPeek 2008; Rabosky 2009).

With lizard lineages in Arid Australia, Rabosky et al. (2011) demonstrated that accounting for lineages in community assembly may readily reveal the ecological traits associated with habitat preferences. In this context, other plausible explanations may be linked to key aspects of the species’ ecology given the intricate nature of the Alps. For example, monocot lineages (i.e., Poaceae, Cyperaceae, and Juncaceae) exhibited more phylogenetically clustered communities (Fig. 2), which may result from the ecological adaptations of these species in the study area, such as dominance in both dry and wet areas. Along the elevation range, monocot species persist in areas of intense competition for light and constitute the dominant biomass in warmer environments. This is likely facilitated by an efficient anemogamous pollination system for pollen movement (Billings 1974; Pellissier et al. 2010a), the inherent physiological capacity to alter patterns of resource allocation (Welker and Briske 1992; Körner 2003), drought-tolerance mechanisms, and the ability to withstand grazing, or even reproduce under high grazing pressure (Körner 2003).

Species of the genus *Carex* (Cyperaceae) are relatively resistant to low temperatures (Körner 2003) and can be dominant and diversified in communities above the treeline, and in moist habitats (Grabherr 1989; Körner 2003). In other different systems, distinct phylogenetic patterns among monocots that differ from coexisting dicots have been reported (Silvertown et al. 2001; Cahill et al. 2008; Mayfield et al. 2009). For instance, Mayfield et al. (2009) demonstrated that dispersal and pollination mechanisms linked to environmental filtering were a prominent process in patterns of phylogenetic clustering among the understory monocots of a fragmented rainforest in Costa Rica. Similarly, from five separate pot experimental studies based on target-competitor combinations, Cahill et al. (2008) reported that the mean phylogenetic distances among monocots showed higher correlated values in comparison with eudicots, signifying a greater tendency toward phylogenetic clustering. In contrast, although a low overlap was observed between coexisting monocots and eudicots in the hydrological niche space reported by Silvertown et al. (2001), a higher pervasiveness of phylogenetic overdispersion was observed among monocots in one of the two studied sites with higher nutrient-rich mineral soil.

A similar trend in the monocots was found among Fabaceae species, which also showed higher prevalence of phylogenetic clustering in communities. Most Fabaceae species are found in warm conditions, except for more basal genera such as *Onobrychis*, *Hedysarum,* and *Oxytropis* that have likely evolved tolerances to colder environments (Körner 2003). The Fabaceae family is largely associated with several symbiotic interactions with fungi and rhizobium bacteria in nitrogen fixation (Mack and Rudgers 2008) and with highly specialized pollinators (often bees, Westerkamp and Claßen-Bockhoff 2007). This feature may constrain their occurrence to communities in more productive eutrophic conditions (because of the significant amounts of nitrogen they produce), although they can be found in extremes of nutrient conditions. The prevailing clustering in the plant families discussed above was in contrast to the prevailing phylogenetic overdispersion in the Asteraceae, Brassicaceae, Campanulaceae, and Polygonaceae species. While Brassicaceae species, for instance, are more ecologically adapted to living in colder environments (Körner 2003), Polygonaceae species may be more restricted to competition-dominated communities at lower or intermediate elevations (see Aeschimann et al. 2004).

Based on the standard values of NRI from this study (including the consideration of ±0.5 values), we found prevailing patterns of phylogenetic overdispersion in the Apiaceae and Lamiaceae, indicating that closely related species diversified into occupy different communities in contrasting environmental conditions, or close relatives co-occur less often than expected. The observed assembly patterns of these groups may in part be attributed to their life-history traits. For instance, Apiaceae species are characterized by heavy diaspores (Körner 2003), which likely provide them with a competitive advantage to establish in communities where their propagules are dispersed, mirroring the competition–colonization trade-off (Levins and Culver 1971). Lamiaceae species have evolved phenolic compounds that provide strong herbivore resistance, and facilitate the persistence of these species in communities in contrasted environments (Grøndahl and Ehlers 2008).

### Does identifying lineage-specific phylogenetic patterns depend on lineage age?

The strength of the observed patterns at phylogenetic nodes may depend on the age of species divergence in the various angiosperm families (Fig. 4). Nodes that are found farther in time along the phylogeny show an increasing tendency toward clustering in the phylogenetic patterns of assembly (Swenson et al. 2006). This was strongly reflected by the more clustered patterns of community assembly among older nodes, whereas more evident opposite phylogenetic patterns were associated with recent divergence events (or younger nodes higher in the tree). This suggests that older lineages likely retain environmental tolerances to occupy communities in similar environments (Hardy and Senterre 2007; Graham et al. 2009). Overall, there was a higher tendency for more recently diverged lineages to display strong phylogenetic patterns than for the older diverged groups, for example from Polygonaceae (5.3 Mya) and Rosaceae (37.2 Mya), respectively. This result supports the conclusions of Hardy and Senterre (2007) and Mayfield et al. (2009) where deep and shallow lineages of rainforest trees exhibited apparent differences in phylogenetic patterns.

Our results further illustrate why studies with focus on a genus or sister groups within a family more readily detect phylogenetic patterns, because genetic variations among more closely related taxa have a likely stronger ecological basis (Cavender-Bares and Wilczek 2003; Losos 2008). For instance, more distinct patterns of phylogenetic overdispersion was found in schoenoid sedges of the Cape Floristic Region of South Africa (Slingsby and Verboom 2006), in *Quercus* species in Florida (Cavender-Bares et al. 2004), and phylogenetic clustering in 28 rainforest tree plots in Borneo (Webb 2000). Thus, the age of a group and the average time since divergence (from a most recent common ancestor) most likely influences the inference that can be drawn from studying the phylogenetic structure of communities in a lineage. Ideally, the measurement of intraspecific trait variation on the field should provide additional insights into how stronger conservatism in deep nodes can be reconciled with the phylogenetic divergences in recent nodes. However, such data are rarely available or partly inconsistent with the main drivers of community assembly.

### Does community phylogenetic signal vary along elevation?

Opposing assembly mechanisms may be nested along an elevation gradient, creating phylogenetic clustering, and overdispersion patterns that are scale independent (e.g., Graham et al. 2009; Swenson and Enquist 2009). Most of the observed trends between NRI and elevation imply that the consequences of species divergence on community structure also depends on prevailing environmental conditions; so that NRI should vary from clustering to overdispersion across an environmental gradient when ecological characters are relatively conserved across lineages (Webb et al. 2002). Elevation and associated environmental conditions typically drive more clustering among species with higher tolerance to conditions at higher elevation (Hardy and Senterre 2007; Bryant et al. 2008; Graham et al. 2009). In our study, we found that the most clustered lineage communities were on average situated above 600 m, whereas the most overdispersed lineage communities began from 400 m, on average (Table S2). Similarly, Wang et al. (2012) found that phylogenetic clustering among microbes increased with elevation due to lower temperatures and frequent temperature fluctuations associated with higher elevations. However, at lower or intermediate elevation, warmer environmental conditions enhance the capacity for increased species interactions and phylogenetic overdispersion (Graham et al. 2009). Good examples here are the hummingbirds of Ecuador (Graham et al. 2009) and the bee communities in the Alps of Germany (Hoiss et al. 2012).

The highly contrasted environmental gradients in the study area may explain in part, the prevailing absence of assembly trends across the phylogenetic tree. This also suggests that the distribution of plant species is influenced by the heterogeneous nature of the environment. This is not surprising as there is a higher tendency for older nodes to contain at least one lineage that radiated at high (e.g., Cyperaceae, Campanulaceae, Saxifragaceae, Gentianaceae) and at low elevation (e.g., Poaceae, Fabaceae). For most plant lineages, we did observe a trend toward decreasing NRI with elevation (Fig. 3), but it is likely that this emanates from the observed gradual decrease in species richness with increasing elevation. Our analyses also showed a higher decrease in NRI with increasing elevation among deeper phylogenetic lineages, in comparison with a subsequent increase in more recently diverged lineages. This pattern was strongly represented in ancient graminoid nodes, and ancient nodes between Fabaceae and Rosaceae species. Within the Cyperaceae, NRI showed an overall increase in communities with increasing elevation, possibly because this lineage is more abundant and thus more species rich in the colder and moister conditions at high elevation. While we found no overall significant influence of species richness on NRI at phylogenetic nodes (see Fig. S4), we acknowledge that the variation in the species richness of lineages may be a methodological limitation (Webb 2000) influencing patterns of NRI along elevation. Hence, better community indices that explicitly incorporate the estimation of species richness might improve the analyses of phylogenetic community structure.

### Caveats in applying a lineage-based framework in community phylogenetics

Despite the advantage of a multiscale analysis of variation in communities, the lineage-specific approach has some important limitations that deserve mention. First, phylogenetic relationships may not reflect the ecological differences among groups of species that diverged under entirely different ecological conditions (Losos 2008). This may arise from species that behave idiosyncratically and render taxon membership a flawed guide to ecological behavior (Silvertown et al. 2001). Phylogeny may therefore be weak, or an inadequate “proxy” for detecting assembly signals under such circumstances (Weiher and Keddy 1999). Second, interpretation of community structure from nodal distances depend on the number and identity of species from a given lineage, such that the resulting range of values within the data set may be biased toward species-rich groups (Webb 2000; Parra et al. 2010). Future studies could account for the overall balance between species-rich and species-poor groups in driving intraspecific phylogenetic richness across lineages. Third, with respect to statistical data analysis, the correlation between phylogenetic patterns and node age could inevitably be an artifact of NRI at different scales, if the type of null model considered, or unmeasured complex properties of the species pool positively influence NRI (Kembel and Hubbell 2006; Kraft et al. 2007). Nevertheless, a time-calibrated phylogeny coupled with estimates of trait evolution should greatly enhance the strong detection of phylogenetic patterns in communities (Kraft et al. 2007; Wiens et al. 2010).

## Conclusions

The node-by-node examination of phylogenetic patterns across mountain plant communities proved more informative than a treewide global analysis. Above all, the detailed analysis of community assembly patterns at phylogenetic nodes revealed a rather weak relationship between the phylogenetic relatedness and ecological similarity of species at several nodes. However, it did show that older phylogenetic lineages tended to be clustered in distinct communities under broad environmental conditions, while more recent nodes may have retained some level of ecological diversification into contrasted conditions.

Detecting these trends required a lineage-specific approach (Wiens and Graham 2005; Hardy and Senterre 2007; Mayfield et al. 2009; Vamosi et al. 2009). Our study proposes a novel framework to unmask subtle transitions in assembly patterns by analyzing phylogenetic patterns separately for different lineages. Considerable evolutionary time appears to be important for revealing patterns of phylogenetic community structure. Altogether, we highlight crucial areas requiring profound scrutiny by future studies, such as the phylogenetic niche conservatism principle, which is constantly challenged when no phylogenetic pattern is detected in a pool of closely related species. The careful disentangling of community assembly patterns permitted better interpretation of community assembly as regards the ecological role of traits among lineages in communities, depicted by transitions, rather than a general conclusion on community structure.

## Supporting Information

**Figure S1.** Molecular dated phylogenetic tree of 231 angiosperm species from the Western Swiss Alps.

**Figure S2.** The relationship between species co-occurrences and phylogenetic distances in (A) monocots (*r* = −0.08, NS) and (B) eudicots (*r* = −0.01, NS) was not significantly different from random.

**Figure S3.** The relationship between species co-occurrence and phylogenetic distances in each vegetation zone along elevation: (A) Colline (*r* = 0.004, NS), (B) Montane (*r* = −0.004, NS), (C) Subalpine (*r* = 0.01, NS), and (D) Alpine (*r* = −0.003, NS) was not significantly different from random.

**Figure S4.** The relationship between net relatedness index and the pool species richness from the most ancestral node.

**Figure S5.** The variation in lineage-specific patterns of community assembly summarized across 100 randomly sampled trees from the posterior distribution of calibrated trees.

**Table S1.** Table of species, genes, and GenBank accession numbers for the additional sequences used in this study.

**Table S2.** Table of the 230 phylogenetic tree nodes with the number of descendent species, the number of plots, and the elevation range at each node.
