# Supplementary figures and images for: Phylogenetic plant community structure along elevation is lineage specific
Source: Ecol Evol. 2013 Nov 8;3(15):4925–39. doi: 10.1002/ece3.868 (PMC3892358; doi:10.1002/ece3.868)

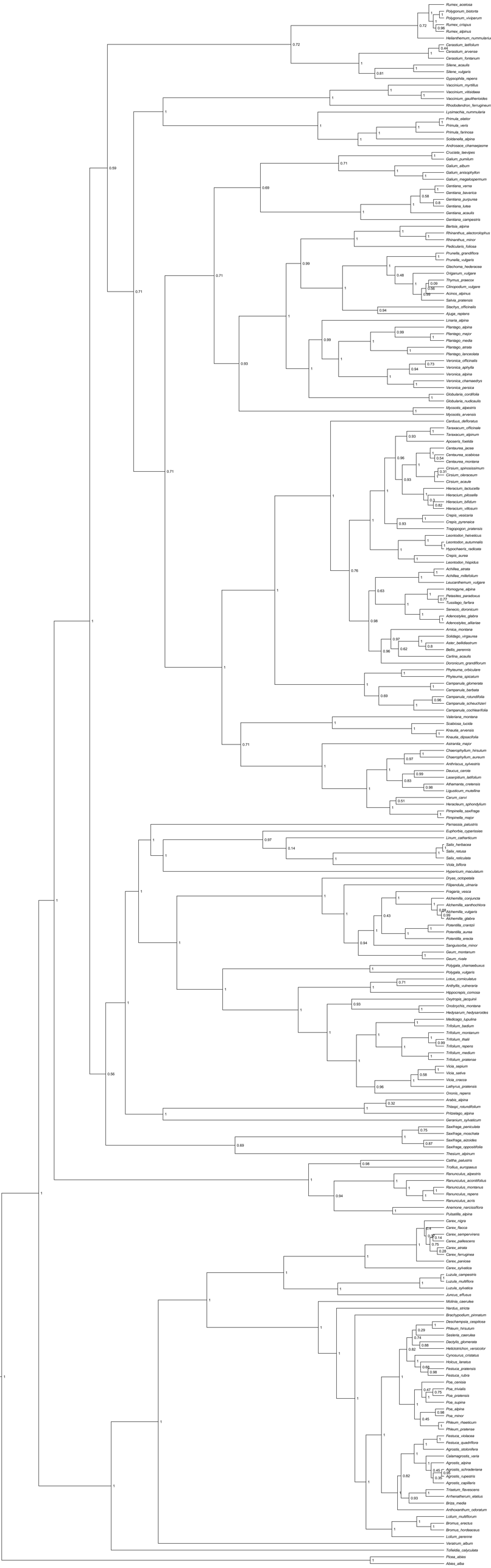

Supplement: Supplementary file 1 [file ece30003-4925-SD1.pdf]

## Monocot phylogenetic structure

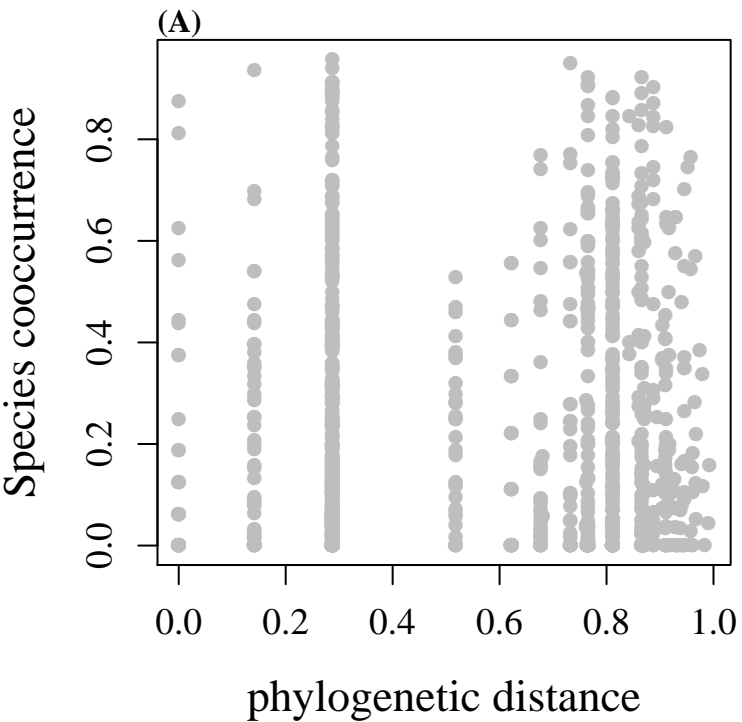

## Eudicot phylogenetic structure

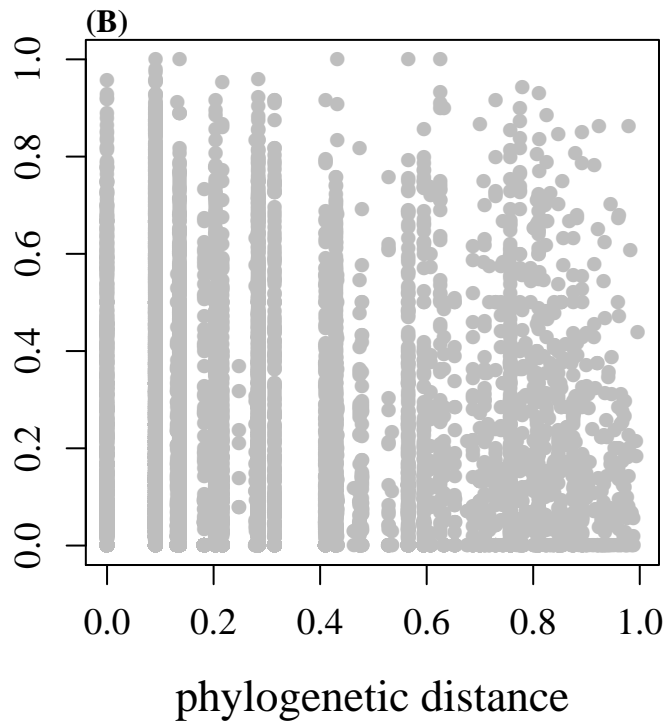

Supplement: Supplementary file 2 [file ece30003-4925-SD2.pdf]

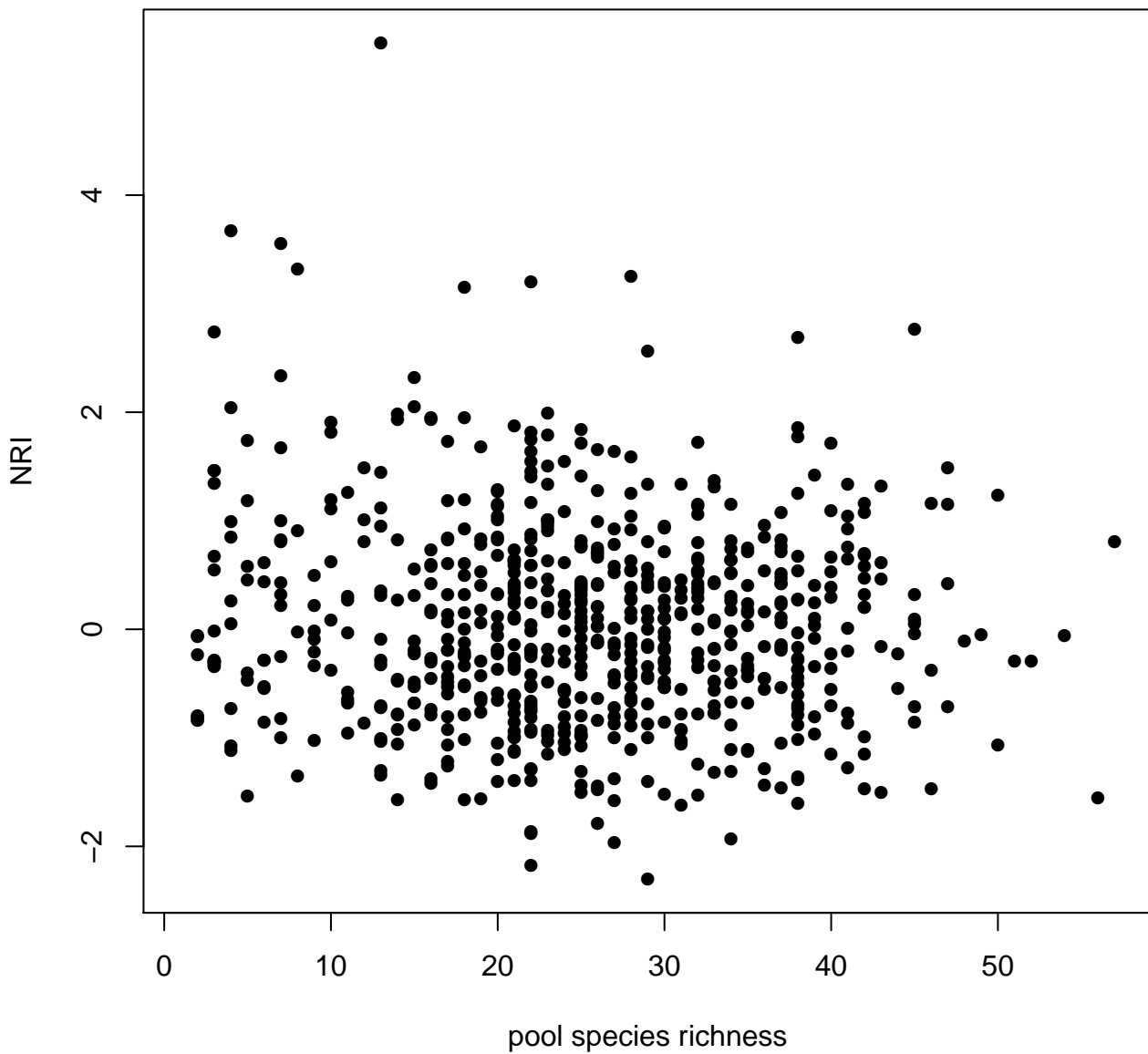

Supplement: Supplementary file 4 [file ece30003-4925-SD4.pdf]

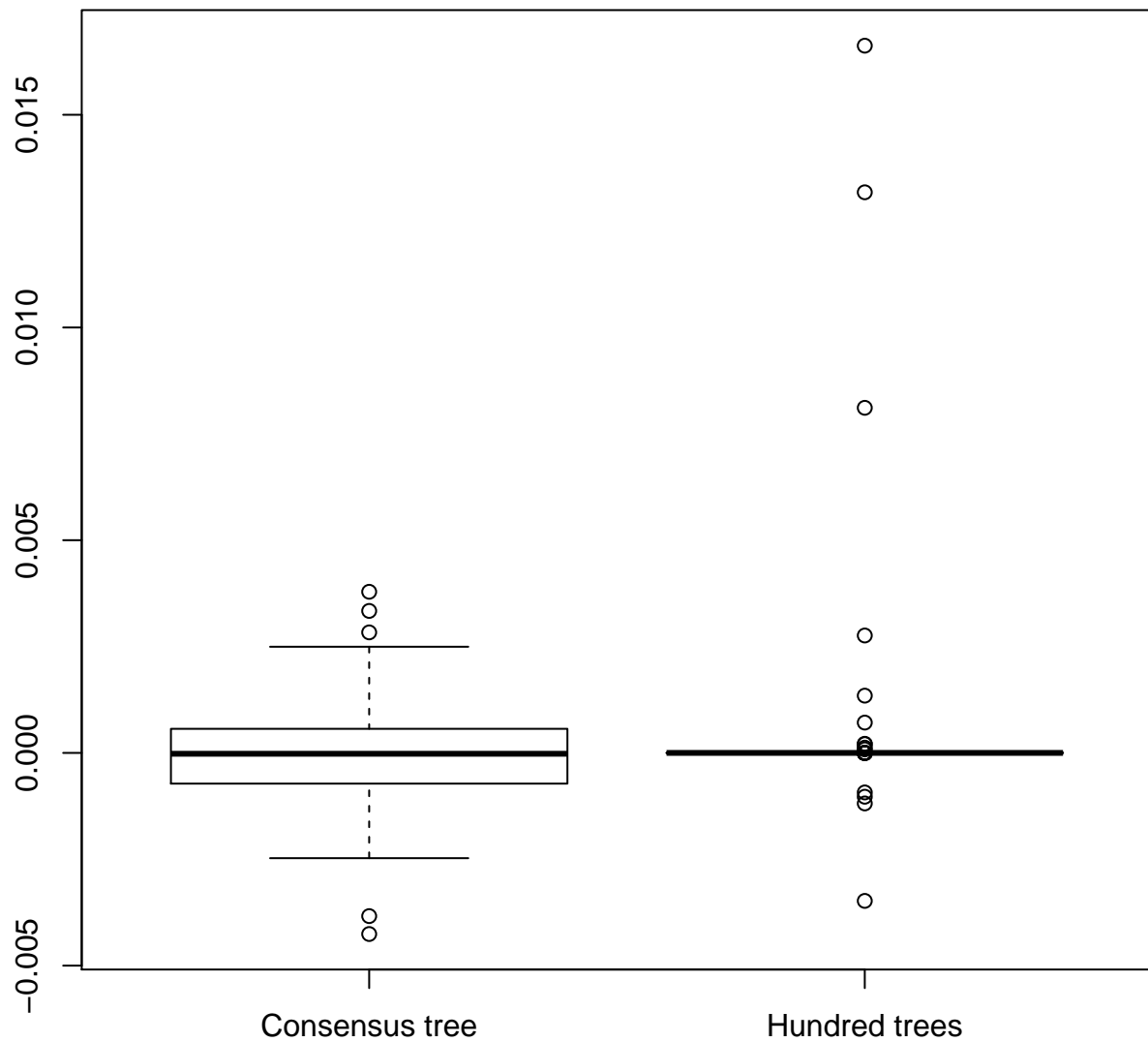

Supplement: Supplementary file 5 [file ece30003-4925-SD5.pdf]
