# Supplementary material for: Phylogenetic plant community structure along elevation is lineage specific
Source: Ecol Evol. 2013 Nov 8;3(15):4925–39. doi: 10.1002/ece3.868 (PMC3892358; doi:10.1002/ece3.868)
Supplement: Supplementary file 3 [file ece30003-4925-SD3.pdf]

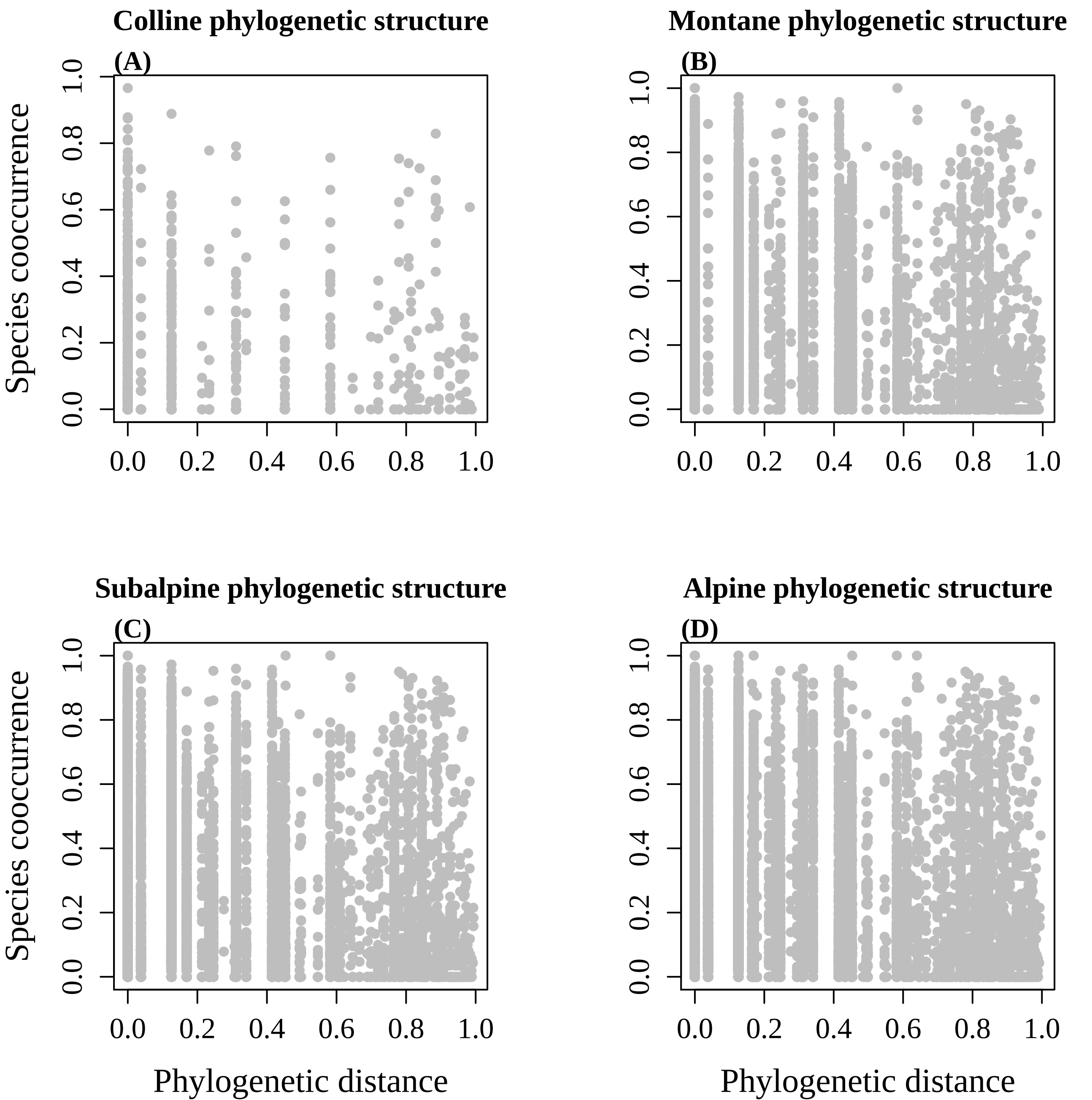
Colline phylogenetic structure
(A)
Montane phylogenetic structure
(B)
Subalpine phylogenetic structure
(C)
Alpine phylogenetic structure
(D)
Species cooccurrence
Phylogenetic distance
0.0
0.2
0.4
0.6
0.8
1.0
