# Supplementary material for: Phylogenetic plant community structure along elevation is lineage specific
Source: Ecol Evol. 2013 Nov 8;3(15):4925–39. doi: 10.1002/ece3.868 (PMC3892358; doi:10.1002/ece3.868)
Supplement: Supplementary file 6 [file ece30003-4925-SD6.pdf]

Table S1 Species, genes and GenBank accession numbers for the additional sequences used in this study.

Species	Family	GenBank Accession # (*RbcL*)	Reference	GenBank Accession # (*MatK*)	Reference
Anthriscus sylvestris (L.) Hoffm.	Apiaceae			ASU58547	Plunkett *et al.* 1996
Carum carvi L.				CCU58553	Plunkett *et al.* 1996
Daucus carota L.		AF207689	Sato *et al.* 1997	DCU58559	Plunkett *et al.* 1996
Laserpitium latifolium L.				LLU58566	Plunkett *et al.* 1996
Achillea millefolium L.	Asteraceae	L13641	Kim *et al.* 1992	EU385315	Panero & Funk 2008
Bellis perennis L.		AY395530	Silvertown *et al.* 2006	HM850613	Schaefer *et al.* 2011
Carduus defloratus L.				AY785091	Susanna (Unpublished)
Crepis aurea (L.) Cass.				EU363564	Enke & Gemeinholzer 2008
Crepis vesicaria L.				AJ633143	Gemeinholzer & Bachmann (Unpublished)
Heracleum sphondylium L.		AY395540	Silvertown *et al.* 2006	FJ395398	James *et al.* (Unpublished)
Hieracium lactucella Wallr.				AJ633170	Fehrer *et al.* 2007
Homogyne alpine (L.) Cass.				GU817473	Pelser *et al.* 2010
Hypochaeris radicata L.		AY395542	Silvertown *et al.* 2006	AJ633231	Gemeinholzer & Bachmann (Unpublished)
Leontodon autumnalis L.				AJ633222	Gemeinholzer & Bachmann (Unpublished)
Leontodon helveticus Mérat				DQ451703	Samuel *et al.* 2006
Leontodon hispidus L.		AY395545	Silvertown *et al.* 2006	AJ633218	Gemeinholzer & Bachmann (Unpublished)
Leucanthemum vulgare Lam.		AY395546	Silvertown *et al.* 2006	FJ395389	James *et al.* (Unpublished)
Pimpinella saxifraga L.		U50229	Plunkett *et al.* 1996	PSU58576	Plunkett *et al.* 1996
Taraxacum alpinum Hegetschw.				AJ633161	Gemeinholzer & Bachmann (Unpublished)
Taraxacum officinale (L.) F.H. Wigg		AY395562	Silvertown *et al.* 2006	AJ633157	Gemeinholzer & Bachmann (Unpublished)
Tragopogon pratensis L.		AY395563	Silvertown *et al.* 2006	FJ395443	James *et al.* (Unpublished)
Tussilago farfara L.				EF537951	Pelser *et al.* 2007
Arabis alpina L.	Brassicaceae			AF144328	Koch *et al.* 2001
Myosotis alpestris L.	Boraginaceae			AY092860	Winkworth *et al.* 2002

Myosotis arvensis Hill.		EU599847	Mansion *et al.* (Unpublished)	EU599671	Mansion *et al.* (Unpublished)
Campanula cochleariifolia Lam.	Campanulaceae			JN571946	Wendling *et al.* 2011
Campanula rotundifolia L.		EU713443	Haberle *et al.* 2009	JN571959	Wendling *et al.* 2011
Campanula scheuchzeri Vill.				JN571962	Wendling *et al.* 2011
Phyteuma spicatum L.		EU643712	Roquet *et al.* 2009	EU713254	Haberle *et al.* 2009
Cerastium arvense L.	Caryophyllaceae			FJ404828	Harbaugh *et al.* (Unpublished)
Cerastium fontanum Baumg.				FJ404829	Harbaugh *et al.* (Unpublished)
Gypsophila repens L.				AY936326	Fior *et al.* 2006
Silene acaulis (L.) Jacq.				EF547235	Mower *et al.* 2007
Silene vulgaris (Moench) Garcke				EF547245	Mower *et al.* 2007
Carex nigra (L.) Reichard	Cyperaceae	GQ469838	Escudero *et al.* 2009	FN668463	Sonstebo *et al.* (Unpublished)
Rhododendron ferrugineum L.	Ericaceae			AB012741	Kurashige *et al.* 1998
Vaccinium myrtillus L.		AY145447	Kron *et al.* 2002	AF382810	Kron *et al.* 2002
Vaccinium vitis-idaea L.		AF419837	Kron 2001	AF382819	Kron *et al.* 2002
Anthyllis vulneraria L.				AF543845	Lavin *et al.* 2003
Euphorbia cyparissias L.	Euphorbiaceae			EU659787	Kim & Kim (Unpublished)
Lathyrus pratensis L.	Fabaceae	AY395544	Silvertown *et al.* 2006		
Lotus corniculatus L.		U74213	Doyle *et al.* 1997	HM049505	Gao *et al.* 2010
Medicago lupulina L.		AY395551	Silvertown *et al.* 2006	HM159569	Steele *et al.* 2010
Onobrychis Montana DC.				AY386879	Wojciechowski *et al.* 2004
Trifolium pratense L.		AY395564	Silvertown *et al.* 2006	HQ619811	Ferri *et al.* (Unpublished)
Trifolium repens L.				AF522131	Steele & Wojciechowski (Unpublished)
Trifolium thalii Vill.				AF522136	Steele & Wojciechowski (Unpublished)
Vicia cracca L.		AY395566	Silvertown *et al.* 2006		
Vicia sativa L.				AF522160	Steele & Wojciechowski (Unpublished)
Gentiana acaulis L.	Gentianaceae			EF552125	Haemmerli & Kuepfer (Unpublished)
Gentiana bavarica L.				EF552079	Haemmerli & Kuepfer (Unpublished)
Gentiana purpurea L.				AJ429323	Bremer *et al.* 2002
Gentiana verna L.		DQ660644	Simoes *et al.* 2007	EF552112	Haemmerli & Kuepfer (Unpublished)
Globularia cordifolia L.	Globulariaceae	AF124558	Olmstead *et al.* 2001	AJ429343	Bremer *et al.* 2002

Globularia nudicaulis L.				AY667473	Rahmanzadeh *et al.* 2005
Juncus effusus L.	Juncaceae	AY216612	Drabkova *et al.* 2006	AB088803	Tamura *et al.* 2004
Luzula campestris (L.) DC.		AY395548	Silvertown *et al.* 2006	HM850953	Schaefer *et al.* 2011
Luzula multiflora (Ehrh.) Lej.		AJ419945	Bremer 2002	HM850955	Schaefer *et al.* 2011
Ajuga reptans L.	Lamiaceae	U32163	Wagstaff & Olmstead (Unpublished)	AY840130	Braeuchler *et al.* 2005
Clinopodium vulgare L.				AY840153	Braeuchler *et al.* 2005
Glechoma hederacea L.		L14292	Olmstead *et al.* 1993	AY840143	Braeuchler *et al.* 2005
Origanum vulgare L.		Z37427	Kaufmann & Wink 1994	GU381802	Braeuchler *et al.* 2010
Prunella grandiflora (L.) Scholler		Z37431	Kaufmann & Wink 1994		
Prunella vulgaris L.		AY395556	Silvertown *et al.* 2006	FJ395426	James *et al.* (Unpublished)
Salvia pratensis L.		AY570436	Walker *et al.* 2004		
Stachys officinalis (L.) Trevis		AF502015	Lindqvist & Albert 2002		
Veratrum album L.		D28168	Kato & Kawano 1995	JF807687	Kim *et al.* (Unpublished)
Linum catharticum L.	Linaceae	FJ169570	McDill *et al.* 2009	HM544103	McDill & Simpson (2011)
Tofieldia calyculata (L.) Wahlenb.	Melanthiaceae	AB183410	Tamura *et al.* (Unpublished)	AB541028	Tamura *et al.* 2010
Bartsia alpina L.	Orobanchaceae	AF190903	Olmstead *et al.* 2001	AY849600	Young & dePamphilis 2005
Pedicularis foliosa L.		AF026836	Wolfe & dePamphilis 1998	AF489959	Leebens-Mack & dePamphilis 2002
Rhinanthus alectorolophus (Scop.) Pollich				AM503829	Li *et al.* 2008
Linaria alpina (L.) Mill.	Plantaginaceae			JF694189	Fernandez-Mazuecos & Vargas 2011
Plantago lanceolata L.		L36454	Olmstead & Reeves 1995	EU718056	Vargas *et al.* 2009
Plantago major L.				EU749326	Fazekas *et al.* 2008
Plantago media L.				AY667474	Rahmanzadeh *et al.* 2005
Veronica chamaedrys L.				FJ395446	James *et al.* (Unpublished)
Veronica officinalis L.		AY034024	Wagstaff *et al.* 2002	HM851037	Schaefer *et al.* 2011
Veronica persica Poir.		AY034022	Wagstaff *et al.* 2002	HM851039	Schaefer *et al.* 2011
Agrostis capillaris L.	Poaceae	AY395527	Silvertown *et al.* 2006	FJ231112	Rotter *et al.* 2010
Agrostis stolonifera L.				FJ231114	Rotter *et al.* 2010
Anthoxanthum odoratum L.		AJ746256	Salamin *et al.* (Unpublished)	AM234541	Doering *et al.* 2007
Arrhenatherum elatius (L.) J. & C. Presl		AJ784823	Salamin *et al.* (Unpublished)	AM234543	Doering *et al.* 2007

Bromus erectus (Huds.) Fourr.				AM234570	Doering *et al.* 2007
Bromus hordeaceus L.		AY395531	Silvertown *et al.* 2006	HM850582	Schaefer *et al.* 2011
Brachypodium pinnatum (L.) P. Beauv.		AM849347	Christian *et al.* 2008	DQ786891	Soreng *et al.* (Unpublished)
Briza media L.				AM234610	Doering *et al.* 2007
Cynosurus cristatus L.		EF125151	Bouchenak-Khelladi *et al.* 2009	DQ786901	Soreng *et al.* (Unpublished)
Dactylis glomerata L.		AY395535	Silvertown *et al.* 2006	AM234595	Doering *et al.* 2007
Deschampsia cespitosa (L.) P. Beauv.		AY691635	Gardner *et al.* (Unpublished)	AM234546	Doering *et al.* 2007
Festuca pratensis Huds.		AY395536	Silvertown *et al.* 2006	HM850535	Schaefer *et al.* 2011
Festuca rubra L.		AJ746261	Salamin *et al.* (Unpublished)	DQ786911	Soreng *et al.* (Unpublished)
Helictotrichon versicolor (Vill.) Schult. & Schult. f				FM957011	Winterfeld *et al.* 2009
Lolium perenne L.		AY395547	Silvertown *et al.* 2006	EU434291	Sungkaew *et al.* 2009
Nardus stricta L.		AY622895	Duvall *et al.* (Unpublished)	AM234573	Doering *et al.* 2007
Molinia caerulea		AY632367	Duvall *et al.* (Unpublished)	AF164411	Hilu *et al.* 1999
Phleum pratense L.		AJ784832	Salamin *et al.* (Unpublished)	DQ786932	Soreng *et al.* (Unpublished)
Poa alpina L.				DQ786933	Soreng *et al.* (Unpublished)
Poa pratensis L.				AF164402	Hilu *et al.* 1999
Poa trivialis L.		AY395555	Silvertown *et al.* 2006	HM850517	Schaefer *et al.* 2011
Sesleria caerulea (L.) Ard.		EF125156	Bouchenak-Khelladi *et al.* 2009	DQ786942	Soreng *et al.* (Unpublished)
Trisetum flavescens (L.) P. Beauv.		AY395565	Silvertown *et al.* 2006		
Polygala chamaebuxus L.	Polygalaceae	AM234197	Forest *et al.* 2007	EU596517	Bello *et al.* 2009
Polygala vulgaris L.		AM234193	Forest *et al.* 2007	EU604046	Bello *et al.* 2009
Polygonum bistorta L.	Polygonaceae	FM883607	Galasso (Unpublished)	AF204859	Meimberg *et al.* 2000
Rumex acetosa L.		AY395559	Silvertown *et al.* 2006	GQ434303	Chen *et al.* 2010
Rumex crispus L.				EU840458	Wang & Liu (Unpublished)
Androsace chamaejasme Wulfen ex Host	Primulaceae			DQ378429	Mast *et al.* 2006
Lysmichnia nummularia L.		AY395550	Silvertown *et al.* 2006		
Primula elatior (L.) Hill				DQ378361	Mast *et al.* 2006
Primula farinose L.				DQ378345	Mast *et al.* 2006
Primula veris L.		AF394982	Trift *et al.* 2002	AJ429293	Bremer *et al.* 2002

Soldanella alpina (L.) F.W. Schmidt				AY647533	Mast *et al.* 2004
Caltha palustris L.	Ranunculaceae	EU053906	Hoot *et al.* 2007	AY515232	Lee & Heo (Unpublished)
Ranunculus aconitifolius L.				AY954217	Paun *et al.* 2005
Ranunculus alpestris L.				AY954221	Paun *et al.* 2005
Ranunculus acris L.				AY954199	Paun *et al.* 2005
Ranunculus montanus Willd.				AY954149	Paun *et al.* 2005
Ranunculus repens L.				HM565166	Emadzade *et al.* 2011
Trollius europaeus				AY515236	Lee & Heo (Unpublished)
Dryas octopetala L.	Rosaceae			JF317424	Zhang *et al.* 2011
Fragaria vesca L.				AF288102	Potter *et al.* 2002
Potentilla erecta (L.) Raeusch.				HM850688	Schaefer *et al.* 2011
Sanguisorba minor Scop.				AB073694	Mishima *et al.* 2002
Salix herbacea L.	Salicaceae			EU790671	Hardig *et al.* (Unpublished)
Salix reticulata L.		AJ235793	Savolainen *et al.* 2000	EU790672	Hardig *et al.* (Unpublished)
Parnassia palustris L.	Saxifragaceae	AY935731	Zhang & Simmons 2006	AY935911	Zhang & Simmons 2006
Saxifraga oppositifolia L.		SOU06217	Soltis *et al.* 1993	SAXCPMA TKD	Johnson & Soltis 1995
Rhinanthus minor L.		AY395558	Silvertown *et al.* 2006		
Valeriana montana L.	Valerianaceae	AY362494	Bell 2004	AY362536	Bell 2004
Viola biflora L.				DQ842607	Yoo & Jang (Unpublished)
		**Outgroup species**			
Abies alba Mill.	Pinaceae	AB029652	Suyama *et al.* 2001 (Unpublished)		
Picea abies (L.) H. Karst.	Pinaceae	AY611034	Bouille & Bousquet 2005		

Table S2 Table of the 230 phylogenetic tree nodes with the number of descendent species, the number of plots, and the elevation range at each node.

Node	No. of species	No. of plots	Elevation range (m)
1	231	693	412.3 – 3041.6
2	181	690	412.3 – 3041.6
3	172	690	412.3 – 3041.6
4	122	677	412.3 – 3041.6
5	12	559	412.3 – 2693.4
6	6	432	412.3 – 2693.4
7	5	392	412.3 – 2693.4
8	4	262	412.3 – 2581.8
9	2	230	412.3 – 2581.8
10	2	40	601.3 – 2500.0

11	6	341	412.3 – 2693.4
12	3	238	412.3 – 2581.8
13	2	59	601.3 – 2500.0
14	3	132	601.3 – 2500.0
15	2	111	601.3 – 2500.0
16	110	662	412.3 – 3017.7
17	10	305	412.3 – 2625.2
18	4	83	601.3 – 2500.0
19	3	82	601.3 – 2500.0
20	2	55	601.3 – 2500.0
21	6	279	412.3 – 2581.8
22	5	269	412.3 – 2581.8
23	4	257	412.3 – 2581.8
24	3	134	601.3 – 2540.0
25	2	127	601.3 – 2500.0
26	100	662	412.3 – 3017.7
27	40	623	412.3 – 2693.4
28	11	396	412.3 – 2693.4
29	5	306	412.3 – 2625.2
30	2	68	601.3 – 2500.0
31	3	258	412.3 – 2581.8
32	2	188	601.3 – 2581.8
33	6	208	412.3 – 2581.8
34	5	176	601.3 – 2581.8
35	4	163	601.3 – 2581.8
36	2	59	601.3 – 2500.0
37	2	113	601.3 – 2500.0
38	29	586	412.3 – 2693.4
39	27	583	412.3 – 2693.4
40	14	436	412.3 – 2693.4
41	4	183	601.3 – 2581.8
42	3	166	601.3 – 2581.8
43	2	86	601.3 – 2500.0
44	10	366	412.3 – 2693.4
45	8	335	412.3 – 2625.2
46	2	253	412.3 – 2581.8
47	6	139	601.3 – 2540.0
48	5	107	601.3 – 2500.0
49	4	103	601.3 – 2500.0
50	3	94	601.3 – 2500.0
51	2	78	601.3 – 2500.0
52	2	134	601.3 – 2540.0
53	13	507	412.3 – 2693.4
54	11	482	412.3 – 2693.4
55	10	468	412.3 – 2693.4
56	5	392	412.3 – 2693.4
57	3	240	412.3 – 2581.8
58	2	124	601.3 – 2500.0
59	2	326	412.3 – 2625.2

60	5	286	412.3 – 2581.8
61	3	77	601.3 – 2500.0
62	2	58	601.3 – 2500.0
63	2	227	412.3 – 2581.8
64	2	40	601.3 – 2500.0
65	2	70	601.3 – 2500.0
66	60	647	412.3 – 2693.4
67	44	639	412.3 – 2693.4
68	37	618	412.3 – 2693.4
69	36	617	412.3 – 2693.4
70	21	564	412.3 – 2693.4
71	16	473	412.3 – 2693.4
72	13	462	412.3 – 2693.4
73	3	326	412.3 – 2625.2
74	2	265	412.3 – 2581.8
75	10	278	412.3 – 2581.8
76	6	167	601.3 – 2581.8
77	3	89	601.3 – 2500.0
78	2	48	601.3 – 2500.0
79	3	91	601.3 – 2500.0
80	2	54	601.3 – 2500.0
81	4	152	601.3 – 2581.8
82	3	76	601.3 – 2500.0
83	2	61	601.3 – 2500.0
84	3	111	601.3 – 2500.0
85	2	77	601.3 – 2500.0
86	5	377	412.3 – 2693.4
87	3	138	601.3 – 2540.0
88	2	93	601.3 – 2500.0
89	2	328	412.3 – 2625.2
90	15	487	412.3 – 2693.4
91	9	443	412.3 – 2693.4
92	3	295	412.3 – 2581.8
93	2	128	601.3 – 2500.0
94	6	247	412.3 – 2581.8
95	3	205	412.3 – 2581.8
96	2	41	601.3 – 2500.0
97	3	73	601.3 – 2500.0
98	2	47	601.3 – 2500.0
99	6	250	412.3 – 2581.8
100	5	235	412.3 – 2581.8
101	4	213	412.3 – 2581.8
102	3	173	601.3 – 2581.8
103	2	149	601.3 – 2581.8
104	7	366	412.3 – 2693.4
105	2	149	601.3 – 2581.8
106	5	320	412.3 – 2625.2
107	2	37	601.3 – 2500.0
108	3	310	412.3 – 2625.2

109	2	254	412.3 – 2581.8
110	16	496	412.3 – 2693.4
111	4	209	412.3 – 2581.8
112	3	196	424.6 – 2581.8
113	2	106	601.3 – 2500.0
114	12	450	412.3 – 2693.4
115	11	443	412.3 – 2693.4
116	7	327	412.3 – 2625.2
117	3	118	601.3 – 2500.0
118	2	101	601.3 – 2500.0
119	4	231	412.3 – 2581.8
120	2	79	601.3 – 2500.0
121	2	155	601.3 – 2581.8
122	4	225	412.3 – 2581.8
123	2	170	601.3 – 2581.8
124	2	131	601.3 – 2500.0
125	50	673	412.3 – 3041.6
126	45	654	412.3 – 2693.4
127	41	614	412.3 – 2693.4
128	8	274	412.3 – 2581.8
129	7	264	412.3 – 2581.8
130	6	219	412.3 – 2581.8
131	5	174	601.3 – 2581.8
132	4	116	601.3 – 2500.0
133	3	91	601.3 – 2500.0
134	2	88	601.3 – 2500.0
135	33	602	412.3 – 2693.4
136	13	529	412.3 – 2693.4
137	12	515	412.3 – 2693.4
138	11	515	412.3 – 2693.4
139	9	511	412.3 – 2693.4
140	8	496	412.3 – 2693.4
141	5	448	412.3 – 2693.4
142	4	441	412.3 – 2693.4
143	3	377	412.3 – 2693.4
144	2	190	601.3 – 2581.8
145	3	286	412.3 – 2581.8
146	2	182	601.3 – 2581.8
147	2	85	601.3 – 2500.0
148	20	554	412.3 – 2693.4
149	2	72	601.3 – 2500.0
150	18	553	412.3 – 2693.4
151	3	360	412.3 – 2693.4
152	2	360	412.3 – 2693.4
153	15	500	412.3 – 2693.4
154	3	52	601.3 – 2500.0
155	2	44	601.3 – 2500.0
156	12	471	412.3 – 2693.4
157	7	463	412.3 – 2693.4

158	2	110	601.3 – 2500.0
159	5	457	412.3 – 2693.4
160	3	356	412.3 – 2693.4
161	2	347	412.3 – 2693.4
162	2	394	412.3 – 2693.4
163	5	198	424.6 – 2581.8
164	4	197	424.6 – 2581.8
165	3	130	601.3 – 2500.0
166	2	87	601.3 – 2500.0
167	4	276	412.3 – 2581.8
168	3	59	601.3 – 2500.0
169	2	36	601.3 – 2500.0
170	5	143	601.3 – 2581.8
171	4	114	601.3 – 2500.0
172	2	58	601.3 – 2500.0
173	2	87	601.3 – 2500.0
174	9	511	412.3 – 2693.4
175	2	125	601.3 – 2500.0
176	7	491	412.3 – 2693.4
177	5	463	412.3 – 2693.4
178	4	435	412.3 – 2693.4
179	3	425	412.3 – 2693.4
180	2	225	412.3 – 2581.8
181	2	72	601.3 – 2500.0
182	50	663	412.3 – 3017.7
183	49	663	412.3 – 3017.7
184	48	663	412.3 – 3017.7
185	12	408	412.3 – 2693.4
186	8	362	412.3 – 2693.4
187	7	351	412.3 – 2693.4
188	6	350	412.3 – 2693.4
189	5	341	412.3 – 2693.4
190	4	306	412.3 – 2625.2
191	2	274	412.3 – 2581.8
192	2	83	601.3 – 2500.0
193	4	155	601.3 – 2581.8
194	3	143	601.3 – 2581.8
195	2	107	601.3 – 2500.0
196	36	662	412.3 – 3017.7
197	35	662	412.3 – 3017.7
198	34	661	412.3 – 3017.7
199	33	661	412.3 – 3017.7
200	29	661	412.3 – 3017.7
201	17	643	412.3 – 2693.4
202	9	578	412.3 – 2693.4
203	5	522	412.3 – 2693.4
204	3	262	412.3 – 2581.8
205	2	147	601.3 – 2581.8
206	2	342	412.3 – 2693.4

207	4	460	412.3 – 2693.4
208	3	456	412.3 – 2693.4
209	2	439	412.3 – 2693.4
210	8	421	412.3 – 2693.4
211	4	177	601.3 – 2581.8
212	3	163	601.3 – 2581.8
213	2	156	601.3 – 2581.8
214	4	293	412.3 – 2581.8
215	2	188	601.3 – 2581.8
216	2	176	601.3 – 2581.8
217	12	589	412.3 – 2693.4
218	11	558	412.3 – 2693.4
219	8	451	412.3 – 2693.4
220	3	162	601.3 – 2581.8
221	2	136	601.3 – 2540.0
222	5	347	412.3 – 2693.4
223	4	310	412.3 – 2625.2
224	3	294	412.3 – 2581.8
225	2	48	601.3 – 2500.0
226	3	267	412.3 – 2581.8
227	2	173	601.3 – 2581.8
228	4	164	601.3 – 2581.8
229	3	111	601.3 – 2500.0
230	2	99	601.3 – 2500.0
